# Time controlled adaptive ventilation™ as conservative treatment of destroyed lung: an alternative to lung transplantation

**DOI:** 10.1186/s12890-021-01545-z

**Published:** 2021-05-22

**Authors:** Malou Janssen, J. Han. J. Meeder, Leonard Seghers, Corstiaan A. den Uil

**Affiliations:** 1 Department of Intensive Care Medicine, Erasmus MC, University Medical Center, Dr Molewaterplein 40, Room Rg 626, 3015 GD Rotterdam, The Netherlands; 2 Department of Pulmonary Medicine, Transplant Center, Erasmus MC, University Medical Center, Rotterdam, The Netherlands; 3 Department of Cardiology, Erasmus MC, University Medical Center, Rotterdam, The Netherlands; 4 Department of Intensive Care Medicine, Maasstad Hospital, Rotterdam, The Netherlands

**Keywords:** Acute respiratory distress syndrome, Bronchopleural fistula, Destroyed lung, Lung protective ventilation, Time-controlled adaptive ventilation, Veno-venous extracorporeal membrane oxygenation

## Abstract

**Background:**

Acute respiratory distress syndrome (ARDS) often requires controlled ventilation, yielding high mechanical power and possibly further injury. Veno-venous extracorporeal membrane oxygenation (VV-ECMO) can be used as a bridge to recovery, however, if this fails the end result is destroyed lung parenchyma. This condition is fatal and the only remaining alternative is lung transplantation. In the case study presented in this paper, lung transplantation was not an option given the critically ill state and the presence of HLA antibodies. Airway pressure release ventilation (APRV) may be valuable in ARDS, but APRV settings recommended in various patient and clinical studies are inconsistent. The Time Controlled Adaptive Ventilation (TCAV™) method is the most studied technique to set and adjust the APRV mode and uses an extended continuous positive airway pressure (CPAP) Phase in combination with a very brief Release Phase. In addition, the TCAV™ method settings are personalized and adaptive based on changes in lung pathophysiology. We used the TCAV™ method in a case of severe ARDS, which enabled us to open, stabilize and slowly heal the severely damaged lung parenchyma.

**Case presentation:**

A 43-year-old woman presented with *Staphylococcus Aureus* necrotizing pneumonia. Progressive respiratory failure necessitated invasive mechanical ventilation and VV-ECMO. Mechanical ventilation (MV) was ultimately discontinued because lung protective settings resulted in trivial tidal volumes. She was referred to our academic transplant center for bilateral lung transplantation after the remaining infection had been cleared. We initiated the TCAV™ method in order to stabilize the lung parenchyma and to promote tissue recovery. This strategy was challenged by the presence of a large bronchopleural fistula, however, APRV enabled weaning from VV-ECMO and mechanical ventilation. After two months, following nearly complete surgical closure of the remaining bronchopleural fistulas, the patient was readmitted to ICU where she had early postoperative complications. Since other ventilation modes resulted in significant atelectasis and hypercapnia, APRV was restarted. The patient was then again weaned from MV.

**Conclusions:**

The TCAV™ method can be useful to wean challenging patients with severe ARDS and might contribute to lung recovery. In this particular case, a lung transplantation was circumvented.

## Background

Acute respiratory distress syndrome (ARDS) is most often caused by severe infection. The pathology is mediated via severe edema and alveolar collapse [1] and can be exacerbated by mechanical ventilation [2, 3]. Gas exchange is often hampered due to damaged lung parenchyma. Besides pharmacological interventions such as surfactant therapy, inhalation of agents contributing to vasodilatation and anti-microbial therapy [4], ventilation strategies are an important factor in the treatment of ARDS. Lung protective ventilation [5] is defined by peak inspiratory pressures less than 30 cm H2O and tidal volumes 4–6 ml/kg/BW, and has been shown to be beneficial in patients with severe ARDS [6]. However, ARDS is still associated with a high mortality rate of about 30–45% [7–9], which allows for the trial of other ventilation strategies. It has been proposed that airway pressure release ventilation (APRV) [10] is applicable in patients with severe ARDS, but the APRV settings reported by various studies and clinical cases [11] are inconsistent. Time-controlled adaptive ventilation (TCAV™) [12]—developed by Dr. Habashi (see attached protocol, ©2021, www.aprvnetwork.org. All rights reserved)—is an APRV protocol that uses extended high plateau pressure times (continuous positive airway pressure, CPAP Phase) in combination with brisk periods of partial pressure release (Release Phase). The very brief Release Phase is set and adjusted based on changes in lung pathophysiology, which is measured by a shift in the slope of the expiratory flow curve (SlopeEF). The SlopeEF curve is a breath-by-breath measurement of respiratory system compliance (CRS). Termination of expiratory flow is calculated by multiplying the peak expiratory flow by 75%, which will determine the Release Phase time. The steeper the SlopeEF, indicating a fall in CRS, the shorter the Release Phase (to prevent derecruitment of alveoli with fast collapse time constants) and the smaller the tidal volume (Vt) (to prevent overdistension of alveolar ducts). As the lung begins to heal and the CRS improves the Release Phase time will increase as will the size of Vt. However, driving pressure will not increase because Vt only increases with an increase in CRS. The TCAV™ method prevents the lung from depressurizing and thereby creates a time controlled positive end-expiratory pressure (TC-PEEP). Alveoli open and collapse as a viscoelastic system and thus the TCAV™ method makes use of the time lag between lung opening after start of inspiration and the time lag between start of expiration and alveolar collapse. The application of continuous positive pressure causes the alveoli to stabilize, and releasing this pressure for a short period of time will not result in alveolar collapse [13]. As a result, this method may prevent ventilator-associated lung injury by averting repetitive alveolar collapse and expansion [14, 15].

The pressure of the CPAP Phase should be set high enough to enable sufficient lung opening and to prevent regional lung strain [16]. Since these pressures are also a function of the time they are applied, longer periods of positive pressures in combination with short expiration periods will result in the maximal opening of alveoli [17]. Since the short expiratory time prevents total expiration, the low-pressure setting is always zero. If these settings are used correctly, APRV might be applied to treat severely damaged lung tissue [18]. The TCAV™ method is designed to rapidly stabilize viscoelastic lung tissue using a very brief Release Phase. Viscoelastic alveoli simply do not have sufficient time to collapse. The extended CPAP Phase will gradually open viscoelastic lung tissue over hours or days, depending on the severity of lung injury when the TCAV™ method is first applied. The end product is a stable and open lung that is highly resistant to VILI-induced injury.

In the case presented here, the lung parenchyma was severely damaged necessitating the use of veno-venous extracorporeal membrane oxygenation (VV-ECMO) to treat the refractory hypoxemia and hypercapnia. VV-ECMO is often used as a bridge until a donor can be found for a lung transplant [19, 20] However, lung transplantation was not possible for this patient, but the TCAV™ method was highly effective at stabilizing and opening this severely injured lung, which eventually allowed for weaning from ECMO and then mechanical ventilation.

## Case presentation

A 43-year-old woman with no remarkable medical history underwent diagnostic arthrography of her right shoulder due to ongoing symptoms following a traumatic injury. Ten days later, the patient was admitted to the same general hospital with *Staphylococcus Aureus* sepsis. Ultrasound examination showed a fluid collection in her right shoulder. Repeated arthrocentesis was performed in order to drain the fluid and antibiotics were started. Three days after admission, she developed respiratory failure for which she was intubated and mechanical ventilation was started. The CT scan showed severe pneumonic infiltrates. Due to further deterioration, the patient was transferred to an academic hospital.

**Fig. 1:**
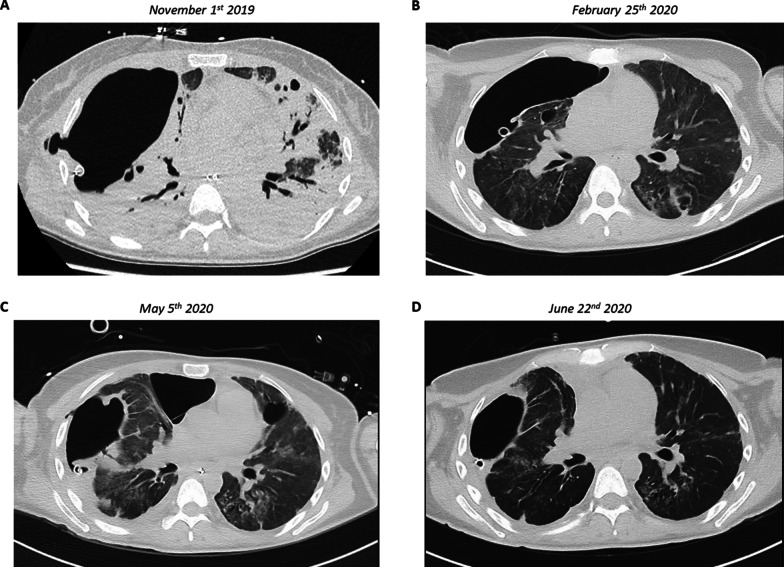
Consecutive CT images showing subsequent phases in the disease course. **a** *CT scan performed in the referring academic hospital*. ECMO, chest tube and tracheostomy tube in situ; the patient was not ventilated, since lung protective settings failed to produce significant tidal volumes. The scan shows bilateral atelectasis, a right-sided bronchopleural fistula, consolidations, destroyed lung parenchyma and a right-sided pneumothorax. **b** *CT scan after ICU discharge and first APRV period*. VV-ECMO has been removed; chest tube and tracheostomy in situ. The APRV mode was set and adjusted by the TCAV™ method with auto-release mode set at 75%. Tidal volumes increased and FiO2 could be decreased during recovery of lung parenchyma. Although the lungs are opened, there are persisting parenchymatic abnormalities of both lungs and persisting air leakage via the bronchopleural fistula. **c** *CT scan after surgery for right bronchopleural fistula closure. C*hest tube and tracheostomy in situ. Pleural effusion is visible after unsuccessful right-sided bronchopleural fistula closure, as well as a persisting pneumothorax. APRV was then restarted. **d** *CT scan after second discharge and APRV period. C*hest tube and tracheostomy in situ. Significant improvement of lung parenchyma is seen after reinstitution of APRV

On transfer the patient was ventilated in the prone position [21]. During the first week, a spontaneous right-sided pneumothorax developed for which a chest tube was placed. Surgical debridement of the right shoulder was performed in order to eliminate the persistent infection. After 7 days she developed refractory respiratory failure and VV-ECMO was initiated. Consecutive CT scans showed a large bronchopleural fistula of the right lung, multiple abscesses and lung parenchyma which at that time was regarded as being destroyed (Fig. 1a). The patient continued to deteriorate and due to the ongoing septic profile, she was referred to our center where the proposal was made to perform a bilateral pneumectomy and then clear the infection, whilst keeping the patient on ECMO until such time as bilateral lung transplantation was possible [22].

At the time of admission to our ICU, 35 days after intubation, the patient was in respiratory distress despite maximum VV-ECMO settings and tracheostomal high-flow oxygen therapy. She was not mechanically ventilated due to the impossibility of achieving adequate tidal volumes with lung protective settings. Successful donor matching for lung transplantation was considered highly unlikely due to the presence of multiple HLA antibodies (Panel-reactive antibody test = 100%). Furthermore, the patient was immobilized and had critical illness polyneuropathy, providing a further contraindication to transplantation. In order to facilitate lung recovery, we applied the TCAV™ method to set and adjust the APRV mode [14, 16] (Dräger Evita® V800). The TCAV™ method applies an extended high pressure CPAP Phase with intermittent very brief Release Phases for CO2 removal. We set the auto-release at 75%, resulting in termination of expiration at 75% of the peak expiratory flow rate, which initially yielded high frequencies and small tidal volumes (9). Our goal was to stabilize the lung with the very short Release Phase to minimize repetitive alveolar collapse-induced atelectrauma. The bronchopleural fistula and corresponding significant air leak persisted and initially worsened, however, because the patient’s collapsed lung parenchyma recruited increasing lung volume, tidal volumes were able to be increased and FiO2 decreased. A tracheostomy was performed eleven days after admission. After a month of APRV and while still on VV-ECMO, her arterial blood gas (ABG) normalized (see Table 1). This strategy enabled the patient to be weaned off VV-ECMO and subsequently mechanical ventilation. After three months the patient was discharged from ICU (Fig. 1b), her tracheostoma had been closed but the chest tube was still in situ.

**Table 1:** Modes and settings of mechanical ventilation and corresponding arterial blood gases (ABG)

	Mode	FiO2/Vti	Pressures (cm H2O)	ABG; pressures in kPa
Admission to ICU at EMC (November 16th)	APRV + VV-ECMO	80% / 2.33 cc/kg 7L FiO2 1.0 sweep gas flow	P_peak_ 41 P_plat_ 40 P_mean_ 28	pH 7.45 pO2 6.8 pCO2 5.7
November 17th	APRV + VV-ECMO	70% / 2.36 cc/kg 7L FiO2 1.0 sweep gas flow	P_peak_ 44 P_plat_ 42 P_mean_ 28	pH 7.49 pO2 11.3 pCO2 5.0
December 20th	APRV + VV-ECMO	40% / 2.72 cc/kg 7.5L FiO2 1.0 sweep gas flow	P_peak_ 36 P_plat_ 32 P_mean_ 23	pH 7.44 pO2 10.3 pCO2 6.6
January 12th	PS + VV-ECMO	30% / 4.34 cc/kg 4L FiO2 1.0 sweep gas flow	P_peak_ 30 P_mean_ 13 PEEP 10	pH 7.38 pO2 11.0 pCO2 8.1
February 25th	No mechanical ventilation, no ECMO	0.5 L O2 nasal cannula	n.a	No ABG
Following surgery and repeat tracheostomy (April 24th)	Start APRV, no ECMO	35% / 4.36 cc/kg	P_peak_ 34 P_mean_ 21 PEEP 11	pH 7.12 pO2 11.2 pCO2 10.5
April 29th	APRV, no ECMO	40%/4.08 cc/kg	P_peak_ 31 P_plat_ 28 P_mean_ 23	pH 7.23 pO2 11.4 pCO2 9.9
May 5th	APRV, no ECMO	30%/3.44 cc/kg	P_peak_ 21 P_plat_ 20 P_mean_ 17	pH 7.41 pO2 11.1 pCO2 6.6
May 31st	APRV, no ECCMO	25%/4.29 cc/kg	P_peak_ 24 P_plat_ 24 P_mean_ 21	pH 7.41 pO2 13.8 pCO2 7.0
June 22nd	No mechanical ventilation, no ECMO	2 L O2 nasal canula	n.a	No ABG

Two months after ICU discharge, surgery was performed to close several remaining bronchopleural fistulas and the patient was readmitted to ICU. Due to drain dysfunction, the fistulas unfortunately reopened. She was extubated the day after surgery, however, due to weakness, atelectasis and ongoing significant air leakage, the patient had to be reintubated after 3 days. One day later, a repeat tracheostomy was performed. Traditional pressure support and control ventilation modes were insufficient and resulted in significant atelectasis so the TCAV™ method was restarted. P_High_ was reduced very slowly. Her clinical situation improved and attending physicians switched to pressure support ventilation, which was regarded as being more comfortable for the patient. However, hypercapnia occurred and after 2 days the patient was exhausted and the TCAV™ method was again restarted. Due to clinical suspicion of infection, antibiotic therapy was broadened. After this infection had been cleared, weaning from ventilation was initiated which was successful after a further 20 days (Fig. 1d).

After less than 2 months, the patient was discharged home with a portable chest tube. The tube dislocated but this did not result in pneumothorax. In October 2020, the patient tested positive for COVID-19, but she only had minor symptoms and there was no need for admission to hospital.

## Discussion and conclusions

In this patient, APRV set with use of the TCAV™ protocol contributed to lung recovery. It enabled us to wean her first from VV-ECMO and then from mechanical ventilation (twice), despite the presence of a large bronchopleural fistula. The TCAV™ method prevented alveolar collapse, which due to lack of surfactant in alveoli easily occurs in lungs affected by ARDS. If the implementation of APRV (both on and off VV-ECMO) earlier on in the course of the disease prevents deterioration and destroyed lung is still only a hypothesis. However, in exceptional cases such as ours, it may be a useful strategy in delaying the indication for lung transplantation.

The beneficial effects of the TCAV™-method have been described in animal models. Using the TCAV™ protocol to set the APRV in Lewis rats, Silva et al. [23] showed that in pulmonary ARDS—but not in extra-pulmonary ARDS—this ventilation strategy was beneficial and resulted in less ventilation-induced injury. Using the same protocol, Roy et al. [24] reported that in pig models with intestinal ischemia and sepsis, that the animals that were ventilated using the TCAV™ method were less likely to develop ARDS than the animals receiving lung protective ventilation. In 2017, Zhou et al. [25] compared the beneficial effects of APRV with auto-release in humans with those of traditional lung protective ventilation. They showed that patients with APRV needed fewer days on mechanical ventilation and were more likely to be extubated. The methodology used in this study closely resembled the TCAV™ protocol, but showed some differences with our strategy because we initially used higher pressures and higher respiratory rates. Recently, the beneficial effects of APRV ventilation have been shown in patients with COVID-19, further emphasizing the relevance of this ventilation mode [26].

The correct settings of this ventilation mode are of great importance, as if Release Phase settings are inadequate they can cause repeated alveolar collapse and corresponding atelectrauma [27]. However, by using the auto-release setting that initiates the stop of expiration at the set percentage of the peak expiratory flow level, alveolar collapse can be prevented. Alternatively, the Release Phase time can be obtained by multiplying the Peak Expiratory Flow by 0.75 to obtain the Termination of Expiratory Flow point. Using the Release Phase time adjustment (Tlow) set the expiratory duration to stop at the calculated Termination of Expiratory Flow value, at which point the lung is rapidly reinflated. In fact, we generated a cast which protected the lungs from ventilation-induced injury. Low tidal volumes were sufficient and increased over time with ongoing tissue recovery.

## Conclusions and directions for further research

In our patient, lungs that were considered beyond repair necessitating a lung transplantation, in fact slowly recovered. In our opinion the TCAV™ method may therefore be a useful tool to aid the recovery of severely damaged lungs. It could even be argued that the severe lung pathology in this patient could have been prevented by initiating the TCAV™ method earlier on in the course of the disease, as suggested by Andrews et al. [28]. Both larger case series and randomized controlled trials are warranted to demonstrate the beneficial effect of the TCAV™ method in different patient groups with ARDS more comprehensively.

## Data Availability

Data is available from the corresponding author upon reasonable request.

## Abbreviations

APRV: Airway pressure release ventilation
ARDS: Acute respiratory distress syndrome
COVID-19: Coronavirus induced disease 2019
CPAP: Continuous positive airway pressure
CRS: Compliance of the respiratory system
CT: Computed tomography
FiO2: Fraction of inspired oxygen
HLA: Human leukocyte antigen
ICU: Intensive care unit
MV: Mechanical ventilation
SlopeEF: Slope of the expiratory flow curve
TCAV™: Time controlled adaptive ventilation
Vt(i): Tidal volume (inspiration)
VV-ECMO: Veno-venous extracorporeal membrane oxygenation
